# Peripherality and Indicators of Nutrition Status in Jewish Israeli Hemodialysis Patients: A Cross-Sectional Study

**DOI:** 10.3390/nu18142222

**Published:** 2026-07-08

**Authors:** Moran Kohavi, Chen Oren Makmal, Nagib Abid, Vered Kaufman-Shriqui, Younes Bathish, Talia Weinstein, Etty Kruzel Davilla, Mona Boaz

**Affiliations:** 1 Department of Nutrition Sciences, Ariel University, Kiryat Hamada 3, Ramat Hagolan St. 36, Ariel 40700, Israel; moranko@ariel.ac.il (M.K.); chenoren24@gmail.com (C.O.M.); nagiba@ariel.ac.il (N.A.); veredks@ariel.ac.il (V.K.-S.); 2 Azrieli School of Medicine, Bar-Ilan University, Safed 1311502, Israel; younes.b@ziv.gov.il (Y.B.); ettyk@gmc.gov.il (E.K.D.); 3 Nephrology Department, Ziv Medical Center, Safed 1311001, Israel; 4 Sackler School of Medicine, Tel Aviv University, Tel Aviv 69978, Israel; tweinste@tauex.tau.ac.il; 5 Medical Center of the Galilee, Nahariya 22100, Israel

**Keywords:** peripherality, malnutrition risk, hemodialysis

## Abstract

**Background:** Geographic peripherality in Israel is linked to poorer health outcomes and may disproportionately affect patients requiring chronic therapies such as hemodialysis (HD). Though malnutrition and inflammation are strong predictors of morbidity and mortality in HD patients, regional differences in nutritional status and dietary adherence are unclear. **Objectives:** To examine the association between peripherality and malnutrition risk, dietary intake, and adherence to nutrition guidelines among Jewish Israeli adults on HD. **Methods:** In this multi-center, cross-sectional study, 154 adult Jewish HD patients were recruited from the northern periphery (*n* = 66) and central regions of Israel (*n* = 88). Demographic, clinical, laboratory, and anthropometric data were obtained from medical records. Nutrient intake was assessed using the multi-pass 24 h dietary recall method. Malnutrition risk was classified using BMI and serum albumin; the C-reactive protein-to-albumin ratio (CAR) was also calculated. Adherence to International Society of Renal Nutrition and Metabolism (ISRNM) dietary guidelines was evaluated. Between-group comparisons and multivariable regression analyses were conducted. **Results:** Overall, participant characteristics were similar between groups; however, coronary heart disease prevalence and dialysis vintage were higher in the periphery. Participants from the periphery had lower serum albumin, blood urea nitrogen, hemoglobin, and blood pressure, but higher LDL cholesterol. Sodium intake was significantly higher and adherence to ISRNM sodium guidelines markedly lower in the periphery. In multivariable analysis, peripherality reduced the odds of meeting sodium recommendations by 92.8%. Adherence to energy and protein guidelines was low in both groups. Nearly half of participants had some level of elevated malnutrition risk using the categorized variable, and an overall difference in the categories of malnutrition risk was detected, driven by the increase in the moderate risk category in the periphery. The composite malnutrition risk variable (any increase in risk vs. no increase in risk) did not differ by peripherality. Peripherality was independently associated with higher percent ideal body weight (%IBW), but not with CAR. **Conclusions:** Peripherality among Jewish Israeli HD patients is associated with differences in nutrition biomarkers, cardiovascular burden, and dietary adherence, especially sodium intake. Interventions considering peripherality should be explored.

## 1. Background

Israel has among the highest life expectancies in the OECD: 83.3–83.8 years [1]. However, this is not uniformly distributed throughout the country. Specifically, individuals who live in the geographic periphery of the country, the north and the south, have lower life expectancy than people who live in the center of the country [2]. Some of this is attributable to differences in the ethnic distribution of the population with a greater percentage of Arabic-speaking ethnic minorities concentrated in the periphery [3]. Due to socioeconomic, lifestyle and genetic factors, these minorities bear a greater burden of many diseases including diabetes, hypertension and cardiovascular disease. But some of the disparity may also be attributable to access to and quality of healthcare services [4], as well as lifestyle factors [5].

Indeed, the geographic distribution of healthcare facilities strongly defines the ability of a given community to access medical services, and this is especially true for therapies required on a chronic basis, such as hemodialysis (HD) [6]. The mortality rate for HD patients who live farther from dialysis centers, such as those in the periphery, is significantly greater than for those who live closer [7].

The early mortality documented in HD patients is influenced by a high burden of comorbidities [8]. In addition, markers of malnutrition risk (reduced serum albumin and elevated C-reactive protein; reduced BMI) are robust predictors of mortality in HD patients [9]. It is not clear whether anthropometric and/or biochemical markers of malnutrition risk, or dietary intake and/or adherence to dietary guidelines, differ by region of residence in HD patients, controlling for ethnicity. Thus, the present study was designed to examine the influence of peripherality on markers of malnutrition risk, nutrition intake and adherence to dietary guidelines among Jewish Israeli adults on maintenance HD therapy.

## 2. Methods

### 2.1. Study Design

This multi-center cross-sectional study included Jewish Israeli adult HD patients treated in the north (Ziv Medical Center, Galilee Medical Center) or the center (Tel Aviv Sourasky Medical Center).

### 2.2. Informed Consent

The study protocol was approved by the Helsinki Committees of the participating dialysis centers: approval no. NHR-0087-22 from Galilee Medical Center in Nahariya, 18 May 2022; approval no. 0062-22-ZIV from Ziv Medical Center in Safed, 13 July 2022; and approval no. 0285-21-TLV from Tel Aviv Sourasky Medical Center in Tel Aviv, 19 May 2021.

### 2.3. Study Population

The study population included all adult (18 years or older) Jewish HD patients, women and men, receiving HD treatment at one of the participating centers, between June 2021 and August 2023. All participants provided signed informed consent prior to enrollment in the study.

### 2.4. Inclusion/Exclusion Criteria

Inclusion in the present study was restricted to Jewish adults receiving maintenance HD at one of the participating HD centers with three months or more dialysis vintage. Declining to provide signed informed consent precluded study participation.

### 2.5. Peripherality

Peripherality was defined by treatment location: north = periphery; Tel Aviv = center.

### 2.6. Demographics, Medical History, Prescribed Medications, Hemodynamic and Laboratory Values

Demographic characteristics, clinical data and prescribed medications were obtained from the electronic medical record. Information on lifestyle characteristics was collected during structured, face-to-face patient interviews.

Systolic and diastolic blood pressure values were calculated as the mean of three post-dialysis measurements: one on the day of the interview and the two most recent prior values.

Laboratory measures included lipid profile, blood count and biochemistry measures. The calcium–phosphorus product was calculated.

### 2.7. Anthropometric Measures

Body weight was calculated as the average of three post-dialysis measurements, including the value on the day of the interview and the two immediately preceding dialysis sessions. Height was extracted from the medical records, and body mass index (BMI) was calculated as mean body weight (kg) divided by height squared (m^2^) [10].

Ideal body weight (IBW) was estimated using sex-specific equations: for men, IBW = 0.9 × height (cm) − 88, and for women, IBW = 0.9 × height (cm) − 92 [11].

### 2.8. Nutrition Intake

Each study participant underwent a structured, in-person multiple-pass 24 h dietary recall interview performed by a clinical dietitian or trained investigator, in accordance with guidelines from the Israel Ministry of Health [12]. These assessments were carried out on dialysis treatment days. Macro- and micronutrient intake was calculated using NutRatio software (https://nutratio.com/).

### 2.9. Adherence to ISRNM Dietary Guidelines

The International Society of Renal Nutrition and Metabolism (ISRNM) provides nutritional recommendations for patients undergoing HD [13] as follows:

- Energy intake: 30–35 kcal/kg IBW per day.
- Protein intake: 1.2–1.4 g/kg IBW per day.
- Sodium intake: 80–100 mmol/day.
- Phosphorus: 800–1000 mg/day.

Intake of these nutrients was calculated from the 24 h recall, which were then dichotomized so that a value of “1” was assigned if the person adhered to the guidelines using the lower value as the cutoffs for energy and protein, and the upper value for the cutoffs for sodium and potassium.

### 2.10. Malnutrition Risk Categories

Malnutrition risk was defined in two ways, both based on the Israeli National Survey of Nutrition in Hemodialysis Patients (SNIPS) [14]. First, using this method, four groups were generated:

- Minimal risk: BMI ≥ 23 kg/m^2^ and serum albumin ≥ 3.8 g/dL.
- Mild risk: BMI < 23 kg/m^2^ and serum albumin ≥ 3.8 g/dL.
- Moderate risk: BMI ≥ 23 kg/m^2^ and serum albumin < 3.8 g/dL.
- Severe risk: BMI < 23 kg/m^2^ and serum albumin < 3.8 g/dL.

Second, a combined variable (“any risk”) grouped all non-minimal categories vs. minimal risk.

### 2.11. C-Reactive Protein to Albumin Ratio (CAR)

The C-reactive protein (CRP) to albumin ratio (CAR), which integrates an inflammatory marker with an indicator of nutrition status, was calculated. CAR ≥ 7 has been identified as a clinically meaningful cutoff for elevated malnutrition risk in this population [15].

### 2.12. Data Quality Assurance

All study data were accessible to the investigators for review. Predefined acceptable ranges were established, and any values falling outside of these limits were cross-checked with the original source records to ensure accuracy.

### 2.13. Participant Privacy

Each participant was assigned a unique study identification code, and all data were anonymized prior to entry into the study database.

## 3. Statistical Methods

### 3.1. Sample Size and Study Power

A sample size of 154 participants provided the present study with 84% power to detect a true difference in serum albumin of no less than 0.2 ± 0.4 g/dL between people receiving HD in the periphery and those treated in the center of the country. This calculation assumes a two-sided 5% alpha.

### 3.2. Data Analysis

All statistical analyses were performed using SPSS software (version 29; IBM Corp., Armonk, NY, USA). The distribution of continuous variables was evaluated using the Kolmogorov–Smirnov test. Variables with a normal distribution are presented as mean ± standard deviation, whereas non-normally distributed variables are summarized as median with interquartile range (IQR). Categorical variables are described as the percentage of participants in each group.

By-region comparisons (periphery vs. center) were conducted using the independent samples *t*-test or the Mann–Whitney U test for continuous variables, as appropriate, based on distribution. Associations between categorical variables were assessed using the chi-square test or Fisher’s exact test when expected cell counts were small.

Separate multivariable binary logistic regression models were attempted for the following endpoints: any malnutrition risk; dichotomized CAR (CAR ≥ 7 vs. CAR < 7); and adherence with each of the ISRNM dietary guidelines (energy, protein, sodium and phosphorus). Odds ratios (ORs) and 95% confidence intervals (CIs) were developed for each predictor. Multivariable linear regression was used to model continuous measures of nutrition status. All models, both logistic and linear, included peripherality (peripheral vs. center). All statistical tests were two-sided, and a *p*-value <0.05 was considered indicative of statistical significance.

## 4. Results

### 4.1. Patient Characteristics

The study population included 154 HD patients: 66 from the periphery and 88 from the center of the country.

In Table 1 the characteristics of the study population are compared by peripherality. Age, sex, current smoking (yes/no), family status number of children, and difficulty with chewing and/or swallowing did not differ by peripherality. No difference in the prevalence of diabetes and hypertension was observed; however, coronary heart disease was significantly more prevalent in participants from the periphery, as were dyslipidemia and history of myocardial infarction. Dialysis vintage was significantly greater in participants from the periphery, but a difference in Kt/V was not detected.

### 4.2. Prescribed Medications

Table 2 presents prescribed medication by peripherality. Most medications were prescribed similarly by peripherality, but phosphate binders and iron supplements were prescribed significantly more frequently, while EPO was prescribed significantly less frequently in the periphery. No between-group difference in the proportion of participants who received either EPO or iron was observed. A regional difference in those who were prescribed both EPO and iron was also not detected. The between-group difference was driven by the greater number of participants who received neither EPO nor iron, which occurred in 51.6% of participants from the periphery but only 33.3% of those from the center.

Only 20% of the study population was prescribed ONS, with no difference between groups. None of the patients in either group received IDPN.

### 4.3. Laboratory and Hemodynamic Measures

Blood chemistry, lipid profile, and hemodynamic measures are compared between participants who live in the periphery and those who live in the center of the country in Table 3. Serum albumin, blood urea nitrogen (BUN), hemoglobin, and the average of the three post-dialysis systolic and, separately, diastolic blood pressure values, were all significantly lower in participants from the periphery than those from the center. With the exception of hemoglobin, these differences persisted even after applying the Bonferroni Correction for multiple comparisons. By contrast, serum low density lipoprotein cholesterol (LDL) was significantly higher in participants from the periphery than the center, though this difference did not persist after applying the Bonferroni Correction.

### 4.4. Nutrient Intake

Table 4 shows the comparison of nutrient intake between participants who live in the periphery and those who live in the center of the country. Participants who live in the periphery consumed significantly more vitamin C and potassium, though intake was below recommended levels in both groups. It should be noted, however, that when the Bonferroni Correction was applied to correct for multiple comparisons, these nutrients no longer significantly differed by peripherality. Additionally, participants from the periphery consumed sodium in excess of ISRNM recommendations, and significantly more than participants from the center, whose intake was within the recommended range. Patients from the center consumed significantly more riboflavin than people from the periphery, but intake was within recommended levels for both groups and no longer differed significantly by peripherality after applying the Bonferroni Correction. By contrast, participants from the center consumed significantly more folic acid than participants from the periphery, but intake levels were below recommended intake values in both groups.

### 4.5. Anthropometry, Malnutrition Risk, and ISRNM Adherence

Table 5 presents the comparison of anthropometric measures, malnutrition risk, and adherence to ISRNM guidelines by peripherality. BMI differed marginally between the groups, and both groups were within the overweight category. There was no by-group difference in the percentage of participants with BMI ≥ 23 kg/m^2^. On the other hand, the percentage of IBW was significantly greater in participants from the periphery than in those from the center of the country.

The distribution of malnutrition risk categories differed significantly by peripherality. Approximately 50% of the participants had minimal malnutrition risk regardless of peripherality. However, an overall by-group difference was observed, driven by a lower percentage of participants in the mild malnutrition risk category and a significantly greater percentage of participants in the moderate malnutrition risk category among people from the periphery. On the other hand, this difference did not persist after applying the Bonferroni Correction.

When the categories were combined into a single measure of elevated malnutrition risk (minimal risk vs. any higher risk), almost half of the participants in both groups had some elevation of malnutrition risk and no between-group difference was detected.

Adherence to ISRNM guidelines was low overall, particularly for energy and protein recommendations. The greatest adherence was noted for phosphorus intake, with no between-group difference. Adherence to sodium intake guidelines was relatively high among participants from the center, more than three times the level of compliance in the periphery, and this difference was significant.

Neither CAR nor the dichotomized CAR variable (CAR ≥ 7) differed by peripherality.

Multivariable binary logistic regression models were attempted for dichotomous endpoints including any malnutrition risk, the dichotomized CAR (CAR ≥ 7) and adherence to each of the ISRNM dietary guidelines. Convergence was achieved only for the model of adherence to the ISRNM guideline for sodium, as shown in Table 6. Periphery was significantly inversely associated with odds of adherence to the guideline, such that being from the periphery significantly reduced the odds of adherence by 92.8%. As universal confounders, age and sex were forced into the model but did not significantly influence odds of this outcome. Dialysis adequacy (Kt/V) significantly improved odds of adherence to the sodium guidelines, such that each 1-unit increase in Kt/V was associated with a more than 16-fold increase in odds of adherence to the guideline; however, it should be noted that the 95% confidence interval for this OR is very wide, potentially suggesting instability of the OR point estimate. The percentage of energy consumed as fat reduced odds of adherence to the guideline, such that each 1% increase in fat as a percentage of total energy intake reduced odds of adherence by 4%. Finally, dietary potassium intake was marginally associated with this outcome, with each 1 mg/day increase in potassium intake associated with a 0.1% reduction in odds of adherence. The model was significant (*p* < 0.001) and correctly classified 79% of study participants for this endpoint.

A multivariable linear regression model was developed to predict %IBW, and the following equation was obtained: %IBW = 192.5 + (13.3 × periphery) − (0.52 × age) − (13.0 × sex) − (27.4 × Kt/V) + (1.93 × dialysis vintage). All predictors in the model were significant: periphery (*p* 0.006); age (*p* = 0.007); sex (*p* = 0.01); Kt/V (*p* < 0.001); and HD vintage (*p* = 0.008). This model was significant (*p* < 0.001) and explained 22% of the variance in %IBW.

A similar model was developed for BUN: BUN = 59.4 − (11.1 × periphery) − (0.03 × age) + (7.9 × sex) − (0.9 × dialysis vintage) + (5.92 × gr protein/kg IBW). In this model, periphery (*p* < 0.001) and sex (*p* = 0.02) were significant, while HD vintage (*p* = 0.062) and gr protein/kg IBW (*p* = 0.09) were marginally significant. The model was significant (*p* < 0.001) and explained 15% of the variability in BUN. Models including peripherality did not converge for BMI or CAR, and a model with peripherality did converge for serum albumin, but it only explained 6% of the variability in this outcome.

## 5. Discussion

The present study identified several clinical, nutritional, and treatment-related differences in HD patients by peripherality. Age, sex, and smoking were similar between groups. Despite this, participants from the periphery had greater prevalence of CHD but not diabetes or hypertension. A greater burden of cardiovascular disease risk has been reported in lower socioeconomic, more peripheral regions compared to more centrally located urban regions across the world [16]. In Israel, the national system of healthcare affords dialysis care to all who require it, but difficulty with transportation logistics may nevertheless make follow-up healthcare access more difficult [7].

Between-group differences in the percentage of the population prescribed EPO, iron supplementation and phosphate binders may reflect systematic by-center differences in disease management; however, it could also reflect a difference in charting practices or reporting. After applying the Bonferroni Correction, there was no by-peripherality difference in hemoglobin levels. Due to the cross-sectional study design, it is not possible to know whether the lack of differences in hemoglobin and serum phosphorus results from or necessitates differences in prescribing practices.

Another between-group difference in participant characteristics was dialysis vintage, which was significantly greater among participants from the periphery. This observation might reflect more limited access to kidney transplantation in the periphery, as has been observed in peripheral populations in other countries, including the United States [17].

Both serum albumin and BUN levels were lower in participants from the periphery, possibly suggesting impaired nutrition status [18], while lower blood pressure readings could reflect poorer hemodynamic stability or differences in antihypertensive management. Low serum albumin, low BUN and low measures of both systolic and diastolic blood pressure have been shown to predict mortality in patients on hemodialysis [19].

Serum LDL cholesterol levels were significantly higher in participants from the periphery, but below 100 mg/dL in both groups. Like in the general population, elevated LDL levels have been associated with increased cardiovascular risk in adults on HD [20]. But in HD patients, the “lipid paradox” has been posited, which predicts that LDL and total cholesterol may decline in hemodialysis as a result of suppressed synthesis in the presence of inflammation, possibly indicating nutritional vulnerability [21]. In HD patients, LDL cholesterol has been shown to have an inverse association with mortality in men, and a U-shaped association in women [22]. There was no by-periphery difference in the percentage of participants prescribed statins.

Intake of specific nutrients differed meaningfully by peripherality. Of particular concern is the significantly increased sodium intake reported by participants from the periphery. In developed countries, salt intake has been reported to be higher in adults from the periphery than from urban centers [23]. This is not necessarily the case in developing countries [24]. High sodium intake may reflect consumption of ultra-processed foods, which—along with other added ingredients—can raise blood pressure, disrupt glycemic control, and are linked to higher rates of hyperphosphatemia, hyperkalemia, and constipation [25]. In HD patients, elevated sodium intake is associated with interdialytic weight gain and serves as a measure of adherence to dietary and fluid restriction; further, excess interdialytic weight gain is associated with increased cardiovascular risk [26].

Consistent with the finding of excess sodium intake, there was significantly lower adherence to sodium recommendations among peripheral patients. This was confirmed in the multivariable analysis and highlights a critical area for intervention. Notably, being from the periphery reduced the odds of sodium guideline adherence by more than 90%, even after adjustment for confounders.

Adherence to the other ISRNM dietary guidelines (energy, protein and phosphorus) was generally low across the cohort, particularly for protein and energy intake, consistent with previous reports in HD populations [14]. Fewer than 15% of participants in the present study adhered to the guideline for energy intake, and approximately 1/3 of participants adhered to the guideline for protein intake, with no difference between the groups. Adherence to the ISRNM dietary phosphorus guidelines was greater than for any other but did not differ by group.

The overall lack of adherence to ISRNM dietary guidelines presents a teaching opportunity for HD center staff, particularly for dialysis nurses. This profession spends the greatest amount of time with HD patients and can leverage this exposure to reinforce dietary messaging. Specifically, nurses, guided by center dietitians, can review general and individualized dietary recommendations to improve compliance. Nurse-led behavioral and educational interventions have been proposed as meaningful ways to promote self-efficacy and improve patient outcomes [27]. Many studies have found that in the general population, overweight and obesity are more prevalent in the periphery than in the urban centers of a country [28]. Participants from the periphery had significantly greater %IBW and marginally greater BMI than those from the center of the country. BMI was elevated and in the overweight range in both groups. Overweight in HD patients, known as the “obesity paradox” posits a survival advantage for overweight and obese patients [29], though some studies have reported that overweight but not obesity status confers protection [30].

In regression analysis, participants from the periphery had a relative 90% reduction in odds of sodium guideline adherence, even after adjustment for confounders. This striking disparity may reflect systematic differences in dietary counseling [26], or health literacy patterns [31]. In patients with congestive heart failure, adherence to dietary sodium restriction was influenced by older age, preference for salty flavors and higher BMI [32]. In this model, each 1-unit increase in Kt/V increased odds of adherence to the ISRNM sodium restriction recommendations by almost 17-fold. It is possible that high sodium intake could increase thirst, leading to greater fluid intake and less adherence to fluid restriction. People on HD who consume more fluids between dialysis sessions often have higher interdialytic weight gain and a larger volume of urea distribution (the V in Kt/V). Increasing the volume of urea distribution can thus lower Kt/V, even if the dialysis dose is unchanged. The association between Kt/V and adherence to sodium restriction is not causative; rather, it is likely a function of fluid retention in non-adherent patients.

Finally, multivariable modeling highlighted the influence of peripherality on %IBW, independent of age, sex, dialysis adequacy, and dialysis vintage. The model suggests that living in the periphery is associated with higher %IBW, even after adjustment for potential confounders, though it explained a modest proportion of variance. Also, differences in macronutrient intake, including by kg/IBW, did not differ by region. Other models, including those for BMI, CAR, and serum albumin, showed limited explanatory power, indicating that additional unmeasured factors—such as individual socioeconomic status, or healthcare access—may play significant roles.

Findings of the present study must be considered in light of its limitations. First, some of the data were collected during times of great stress, including the end of the COVID-19 pandemic, which may have influenced eating patterns in participants. Participants from the periphery are close to the border with Lebanon, and sporadic missile attacks occurred during the data collection period, which may have altered typical dietary intake. Dietary intake was measured using a single 24 h recall. This method is limited by recall bias as well as its inability to capture individual “typical” intake; however, it can represent the mean population intake, though with exaggerated standard deviation. This increases the likelihood of a type II statistical error, where an association in fact exists, but the investigator accepts the null hypothesis [33]. When the nutrient in question is prone to a great deal of daily variability [34], it may compound the problem; nevertheless, the present study did identify differences in sodium intake by peripherality, though it may be an underestimation of the true difference. Because all 24 h recalls were performed on a dialysis day, intake reflects non-dialysis days, which may be different from dialysis days. This may limit external validity such that findings cannot be generalized to dietary intake on dialysis days. Another study limitation is the cross-sectional design of the study, which precludes any consideration of causality. Importantly, the study only includes Jewish Israeli HD patients, due to the genetic, cultural and dietary confounding that would be introduced into the study by including other ethnicities. This creates a limitation on the external validity of the findings, which cannot be generalized to the entire Israeli HD population.

The study also has several strengths. First, limiting participation to members of one ethnicity reduces confounding introduced by differences in cultural, genetic and behavioral patterns, some of which are unmeasurable. The sample size was adequate to detect the between-group differences in peripherality. Also, data collection was performed by highly trained investigators to maximally reduce measurement error.

## 6. Conclusions

In summary, this study identifies important regional disparities among HD patients, with those in the periphery experiencing higher CHD burden, greater %IBW and lower adherence to dietary sodium guidelines. These findings may imply inequities or at least differences in healthcare delivery, nutrition management practices, and patient support. Interventions aimed at improving nutrition counseling, access to appropriate foods, and management of anemia and CHD risk in the periphery may help reduce these disparities and improve patient outcomes.

## Figures and Tables

**Table 1:** Participant characteristics by peripherality.

Characteristic	Periphery N = 66	Center N = 88	*p*-Value
Age (years) *	70 (15.0)	69 (15.8)	0.752
Sex (% female)	33.8	34.1	0.975
Present smoking (%)	16.7	17.0	0.950
Family status			0.415
Married (%)	63.6	51.1	
Widowed (%)	15.2	15.9	
Divorced (%)	10.6	13.6	
Single/other (%)	10,6	19.3	
Number of children *	3 (2)	3 (3)	0.628
Difficulty chewing/swallowing (%)	16.9	10.2	0.225
Comorbidities			
Hypertension (%)	90.8	85.2	0.304
Diabetes (%)	52.3	56.8	0.579
Coronary heart disease (%) **	58.5	38.6	0.015
Myocardial infarction (%)	16.7	5.7	0.03
Dyslipidemia	62.1	30.7	0.001
Dialysis vintage (years)	3.0 (4.0)	2.0 (4.4)	0.006
Dialysis adequacy (Kt/V)	1.4 ± 0.4	1.5 ± 0.3	0.339

* With the exception of Kt/V, which is presented as mean ± standard deviation, all continuous data had distributions that significantly deviated from normal, so are presented as median (IQR). ** Coronary heart disease includes a history of stable or unstable angina; history of myocardial infarction; percutaneous transluminal coronary angioplasty; and coronary artery bypass graft.

**Table 2:** Prescribed medications by peripherality.

Percent of Population with Prescription in Medical Record			
Medication Prescribed	Periphery N = 66	Center N = 88	*p*-Value
Phosphate binders	92.3	70.1	<0.001
Erythropoietin	17.2	58.6	<0.001
Antihypertensive agents	89.4	85.2	0.447
Iron	46.2	29.9	0.043
Folic Acid	65.6	54.0	0.152
B-vitamin Supplements *	36.9	33.3	0.646
Aspirin	55.4	48.3	0.386
Statins	50.8	48.3	0.761
Oral anti-hyperglycemic agents	35.4	44.3	0.266
Diuretics	57.8	47.1	0.194
Oral Nutrition Supplements	24.6	19.3	0.441
IDPN **	0	0	-

* B-vitamin supplements include vitamins B1, B2, B3, B6 or B12, individually or in combination. ** IDPN = intradialytic parenteral nutrition.

**Table 3:** Blood chemistry, blood count, lipid profile and hemodynamic measurements by peripherality.

Measure	Periphery N = 66	Center N = 88	*p*-Value
Blood Chemistry			
Random Glucose (mg/dL)	105.0 (74.8)	115.0 (51.0)	0.358
Albumin (g/L)	3.9 (0.4)	4.0 (0.4)	0.003
C-reactive protein (mg/dL)	7.3 (11.4)	7.1 (14.4)	0.565
Creatinine (mg/dL)	8.1 ± 2.5	7.2 ± 2.1	0.134
Blood urea nitrogen (mg/dL)	54.0 (23.3)	63.8 (25.0)	0.004
Calcium (mg/dL)	8.4 ± 0.7	8.6 ± 0.7	0.602
Phosphorus (mg/dL)	4.9 (1.0)	5.0 (0.6)	0.340
Calcium–Phosphorus product (mg^2^/dL^2^)	40.3 (10.0)	40.9 (17.1)	0.397
Potassium (mmol/L)	5.0 ± 0.7	5.1 ± 0.6	0.465
Blood Count			
Hemoglobin (g/L)	10.5 ± 2.3	11.3 ± 1.2	0.041
White blood cells (10^9^/L)	6.2 (2.0)	6.6 (2.6)	0.820
Lipid Profile			
Total cholesterol (mg/dL)	144.0 (66.0)	118.0 (29.5)	0.328
HDL (mg/dL)	38.0 (17.0)	35.5 (20.1)	0.426
LDL (mg/dL)	78.1 (45.2)	59.0 (28.0)	0.022
Triglycerides (mg/dL)	122.6 (93.3)	115.0 (97.0)	0.204
Hemodynamic Measures			
Systolic blood pressure (mmHg)	134.5 ± 21.5	148.1 ± 19.7	<0.001
Diastolic blood pressure (mmHg)	60.5 (16.3)	71.2 (16.7)	<0.001

Measures with normally distributed measures, including phosphorus, potassium, systolic and diastolic blood pressure are presented as mean ± standard deviation. The remaining measures have distributions significantly deviating from normal and so are presented as median (IQR). HDL = high density lipoprotein cholesterol; LDL = low density lipoprotein cholesterol.

**Table 4:** Nutrient intake compared by peripherality.

Nutrient	Periphery N = 66	Center N = 88	*p*-Value
Energy (kcal/day)	1386.1 (462.9)	1403.3 ± 599.6	0.850
Energy (kcal/kg IBW)	22.5 ± 6.9	21.6 ± 8.7	0.527
Protein (g/day)	63.9 (36.2)	61.6 (42.8)	0.360
Protein (g/kg IBW)	1.1 ± 0.4	1.0 ± 0.5	0.144
Protein (% total kcal)	17.7 (10.3)	17.1 (7.8)	0.200
Fat (% total kcal)	37.2 ± 11.4	36.5 ± 10.1	0.348
Saturated fat (% total kcal)	11.0 (6.5)	10.6 (7.5)	0.706
Carbohydrates (% total kcal)	43.1 ± 10.8	45.2 ± 10.9	0.252
Vitamin A (μg/day)	1773.3 (3013.6)	1244.9 (1709.8)	0.734
Thiamine (mg/day)	0.9 (0.8)	0.9 (0.8)	0.781
Riboflavin (mg/day)	1.2 (0.7)	1.7 (1.7)	0.007
Niacin (mg/day)	16.2 (13.6)	16.9 (12.1)	0.862
Vitamin B6 (μg/day)	1.1 (0.9)	0.9 (1.2)	0.801
Vitamin B12 (μg/day)	2.4 (2.4)	2.4 (3.2)	0.994
Folic acid (μg/day)	78.1 (88.5)	234.4 (194.2)	<0.001
Vitamin C (mg/day)	45.7 (64.9)	35.3 (62.6)	0.031
Calcium (mg/d)	394.5 (410.3)	475.9 (340.0)	0.234
Phosphorus (mg/day)	884.2 (511.6)	803.6 (459.8)	0.290
Potassium (mg/day)	1885.2 (1109.7)	1562.6 (821.4)	0.023
Sodium (mg/day)	2938.4 (2401.2)	1934.5 (1397.1)	<0.001
Iron (mg/day)	6.8 (5.5)	8.2 (6.2)	0.102

The nutrients’ energy (kcal/day), energy (kcal/kg IBW), protein (g/kg IBW), fat (% total kcal), and carbohydrates (% total kcal) had normal distributions so are presented as mean ± standard deviation and compared by peripherality using the *t*-test for independent samples. The remaining nutrients had distributions significantly deviating from normal, and so are presented as median (IQR), and were compared by peripherality using the Mann–Whitney U test. IBW = ideal body weight. Nutrient intakes reflect intake from the diet and not nutritional supplements.

**Table 5:** A comparison of anthropometric measures, malnutrition risk and adherence to ISRNM dietary guidelines by peripherality.

Measure	Periphery N = 66	Center N = 88	*p*-Value
**Anthropometric Measures**			
**Body mass index** (kg/m^2^)	27.4 (9.1)	25.5 (6.2)	0.080
**BMI ≥ 23 kg/m^2^** (%)	81.0%	70.1%	0.132
**Percent Ideal body weight** (%)	130.1 ± 33.2	115 ± 27.1	0.002
**Malnutrition Risk (%)**			
**Minimal** (BMI ≥ 23 kg/m^2^ and albumin ≥ 3.8 mg/dL)	50.8	53.4	0.033
**Mild** (BMI < 23 kg/m^2^ and albumin ≥ 3.8 mg/dL)	7.9	22.7	
**Moderate** (BMI ≥ 23 kg/m^2^ and albumin < 3.8 mg/dL)	30.2	15.9	
**Severe** (BMI < 23 kg/m^2^ and albumin < 3.8 mg/dL)	11.1	8.0	
**Any malnutrition risk (minimal risk vs. any increase in risk)**	49.2	46.6	0.751
**ISRNM nutrition recommendations for hemodialysis patients (%)**			
**Energy (kcal):** 30–35 kcal/kg/day *	13.3	11.4	0.719
**Protein:** 1.2–1.4 g/kg/day *	31.7	31.4	0.972
**Sodium:** 80–100 mmol/day *	19.4	64.8	<0.001
**Phosphorus:** 800–1000 mg/day plus binders if elevated *	64.5	69.3	0.537
**C-reactive protein-to-albumin ratio (CAR)**			
**CAR (mg/gr)**	1.9 (3.1)	1.9 (3.5)	0.586
**CAR ≥ 7 (%)**	15.6	10.4	0.378

* When the ISRNM guidelines are presented as a range, the lower range limit is used as the cutoff for energy and protein, and the upper range limit is used for sodium and phosphorus. ISRNM = International Society of Renal Nutrition and Metabolism.

**Table 6:** Logistic regression model of adherence to ISRNM guidelines for sodium.

Variable	Odds Ratio	95% Confidence Interval of OR	*p*-Value
Periphery (yes = 1, no = 0)	0.072	0.025–0.205	<0.001
Age (years)	0.97	0.934–1.006	0.101
Sex (male = 1, female = 0)	1.068	0.364–3.134	0.904
Dialysis adequacy (Kt/V)	16.877	2.711–105.064	0.002
Fat (% of total energy intake)	0.959	0.938–0.981	<0.001
Dietary potassium (mg/day)	0.999	0.999–1.000	0.05
Constant	10.311		0.188

## Data Availability

The data are not publicly available due to privacy restrictions; however, the data set is available upon reasonable request from the corresponding author.

## References

[1] Organisation for Economic Co-operation and Development (OECD) (2025). Health at a Glance 2025: Country Notes—Israel. https://www.oecd.org/content/dam/oecd/en/publications/reports/2025/11/health-at-a-glance-2025-country-notes_2f94481e/israel_23aa66e2/b3600fbc-en.pdf.

[2] Saabneh A.M. (2016). Arab-Jewish gap in life expectancy in Israel. Eur. J. Public Health.

[3] Muhsen K., Green M.S., Soskolne V., Neumark Y. (2017). Inequalities in non-communicable diseases between the major population groups in Israel: Achievements and challenges. Lancet.

[4] Saabneh A. (2022). Health inequalities between Palestinians and Jews in Israel: The role of extreme spatial segregation. Popul. Space Place.

[5] Israel Ministry of Health (2025). Report on Health Equity: Indices, Gaps and Policy Recommendations 2023 (Hebrew). https://www.gov.il/BlobFolder/reports/health-equity-report-2023/he/files_publications_units_financial-strategic-planning_publications_inequality_health-equality-2023.pdf.

[6] Flythe J.E., Watnick S. (2024). Dialysis for Chronic Kidney Failure: A Review. JAMA.

[7] Namimi-Halevi C., Keinan-Boker L., Dichtiar R., Beckerman P., Bromberg M. (2025). Travel Distance to Dialysis and Mortality Among Hemodialysis Patients in a Geographically Small Country. J. Community Health.

[8] Noh J., Park S.Y., Bae W., Kim K., Cho J.H., Lee J.S., Kang S.W., Kim Y.L., Kim Y.S., Lim C.S. (2024). Predicting early mortality in hemodialysis patients: A deep learning approach using a nationwide prospective cohort in South Korea. Sci. Rep..

[9] Yip W., Ng S.H.X., Kaur P., George P.P., Guan J.H.C., Lee G., Koh T.J.K., Tan W.S., Hum A.Y.M. (2024). Risk factors for short-term all-cause mortality in patients with end stage renal disease: A scoping review. BMC Nephrol..

[10] Hirani V., Mindell J. (2008). A comparison of measured height and demi-span equivalent height in the assessment of body mass index among people aged 65 years and over in England. Age Ageing.

[11] Bonner M., Wright D. (2017). Practical Pharmaceutical Calculations.

[12] Keinan-Boker L., Shohat T., Shimony T., Goldsmith R., Nitsan L. (2019). Rav Mabat Adult Second National Health and Nutrition Survey Ages 18–64 2014–2016. Israel Center for Disease Control (ICDC) Medical Technology, Information and Research Division Ministry of Health. Publication No. https://www.gov.il/BlobFolder/reports/mabat-adults-2014-2016-383/en/files_publications_units_ICDC_mabat_adults_2014_2016_383_en.pdf.

[13] Ikizler T.A., Cano N.J., Franch H., Fouque D., Himmelfarb J., Kalantar-Zadeh K., Kuhlmann M.K., Stenvinkel P., TerWee P., Teta D. (2013). Prevention and treatment of protein energy wasting in chronic kidney disease patients: A consensus statement by the International Society of Renal Nutrition and Metabolism. Kidney Int..

[14] Boaz M., Azoulay O., Schwartz I.F., Schwartz D., Assady S., Kristal B., Benshitrit S., Yanai N., Weinstein T. (2019). Malnutrition Risk in Hemodialysis Patients in Israel: Results of the Status of Nutrition In Hemodialysis Patients Survey Study. Nephrology.

[15] Li M., Jiang Z., Zhang D. (2025). The C-reactive protein/albumin ratio as a nutritional biomarker in maintenance hemodialysis patients: A cross-sectional study of malnutrition-inflammation status assessment. Front. Med..

[16] Global Burden of Cardiovascular Diseases and Risks 2023 Collaborators (2025). Global, Regional, and National Burden of Cardiovascular Diseases and Risk Factors in 204 Countries and Territories, 1990–2023. J. Am. Coll. Cardiol..

[17] Britt-Spells A.M., Buford J., Kelty C., Manjal P., Ross-Driscoll K., Fridell J.A., Pastan S., Patzer R.E., Wilk A.S. (2026). Association Between Rurality and Evaluation Start Among Referred Patients with End Stage Kidney Disease. Am. J. Nephrol..

[18] Asai K., Shibata M., Ito I., Tawada H., Taniguchi S. (2023). Cumulative C-Reactive Protein Levels and Progression of Malnutrition in Dialysis Patients: A Longitudinal Study. Blood Purif..

[19] Spiegel D.M., Raggi P., Smits G., Block G.A. (2007). Factors associated with mortality in patients new to haemodialysis. Nephrol. Dial. Transplant..

[20] Huang J., Li Y., Shen S., Yu L., Sun Q. (2025). Study on causes of death and influencing factors in hemodialysis patients with End-Stage renal disease. BMC Urol..

[21] Takahashi R., Obi Y., Kalantar-Zadeh K., Kovesdy C.P. (2026). The obesity and lipid paradoxes in chronic kidney disease: Mechanisms, interventions, and future directions. Curr. Opin. Nephrol. Hypertens..

[22] Kim S.G., Park E.H., Park W.Y., Cho J.H., Yu B.C., Han M., Song S.H., Ko G.J., Yang J.W., Chung S. (2025). Age and sex-specific association between dyslipidemia treatment and mortality in elderly Korean hemodialysis patients: A retrospective cohort study by the Korean Society of Geriatric Nephrology. Clin. Nephrol..

[23] Nosratinia N., Azadnajafabad S., Masinaei M., Golestani A., Ghamari S.H., Abbasi-Kangevari M., Rezaei N., Khosravi S., Rezaei S., Ahmadi N. (2025). Salt Intake Among the Iranian Population and Public Attitudes Toward Salt Consumption: National and Subnational Report From STEPS 2021. Food Sci. Nutr..

[24] Karmakar N., Datta A., Nag K. (2021). Control of Dietary Salt Intake at Family Level by Housewives in Tripura, India: A Rural—Urban Comparison. Kathmandu Univ. Med. J. (KUMJ).

[25] Padial M., Taylor A., Sabatino A., Piccoli G.B., Avesani C.M. (2024). From ultra-processed foods towards healthy eating for CKD patients: A proposal of educational infographics. J. Nephrol..

[26] Bossola M., Mariani I., Strizzi C.T., Piccinni C.P., Di Stasio E. (2025). How to Limit Interdialytic Weight Gain in Patients on Maintenance Hemodialysis: State of the Art and Perspectives. J. Clin. Med..

[27] Elmansy F.M., Elbqry M.G., Qalawa S.A.A., AlQasem W.A., Alotni M.A., Alharbi M., Shalby A.Y.M. (2026). Nutritional indicators and fluid self-efficacy among hemodialysis patients in the Qassim region, Saudi Arabia: A multicenter descriptive cross-sectional study. Front. Nutr..

[28] Nguyen P.H., Maasen K., Talsma E.F., Zagré R.R., Gune S., Tran L.M., Truong M.T., Hoang N.T., Vuong N.H.T., Fretes G. (2026). Urbanicity and the diets and nutritional status of adolescents and their mothers in Vietnam^1–4^. J. Nutr..

[29] Wathanavasin W., Kaewput W., Thongprayoon C., Tangpanithandee S., Suppadungsuk S., Cheungpasitporn W. (2026). Association of Obesity and Malnutrition with In-Hospital Mortality and Clinical Outcomes in Patients Receiving Maintenance Dialysis: A National Database Study. Nutrients.

[30] Motofelea A.C., Olariu N., Pecingina R., Marc L., Chisavu L., Bob F., Mihaescu A., Apostol A., Schiller O., Motofelea N. (2026). BMI Category and Survival in Incident Hemodialysis Patients: The Overweight Advantage in an Eastern European Cohort. J. Clin. Med..

[31] Schiff R., Freill H. (2020). Improving access to phosphorus- and sodium-restricted foods for people living with chronic kidney disease in remote First Nations. Rural Remote Health.

[32] Chen S., Qiu X., Dai Y., Deng Z., Liu N., Xie R., Qiu X., Zhang L. (2026). Factors influencing sodium restriction behavior among patients with chronic heart failure: A cross-sectional study based on the value co-creation theory. BMC Cardiovasc. Disord..

[33] Foster E., Bradley J. (2018). Methodological considerations and future insights for 24-hour dietary recall assessment in children. Nutr. Res..

[34] Lew S.Q., Asci G., Rootjes P.A., Ok E., Penne E.L., Sam R., Tzamaloukas A.H., Ing T.S., Raimann J.G. (2023). The role of intra- and interdialytic sodium balance and restriction in dialysis therapies. Front. Med..

